# Antinociceptive Activity of* Zanthoxylum piperitum* DC. Essential Oil

**DOI:** 10.1155/2016/3840398

**Published:** 2016-07-31

**Authors:** Graciela Rocha Donald, Patrícia Dias Fernandes, Fabio Boylan

**Affiliations:** ^1^Federal University of Rio de Janeiro, Institute of Biomedical Sciences, Avenida Carlos Chagas Filho 373, CCS, Laboratory J10, 21941-902 Rio de Janeiro, RJ, Brazil; ^2^School of Pharmacy and Pharmaceutical Sciences, Trinity Biomedical Sciences Institute, Trinity College Dublin, Dublin, Ireland

## Abstract

*Zanthoxylum piperitum* DC. (ZP) is a traditional medicinal plant used mainly in countries from Asia such as Japan. This study aimed to investigate the antinociceptive effect of ZP essential oil (ZPEO). The major component present in the essential oil was beta-phellandrene (29.39%). Its antinociceptive activity was tested through animal models (formalin-, capsaicin-, and glutamate-induced paw licking and hot plate). The anti-inflammatory effect was evaluated through the carrageenan-induced leukocyte migration into the subcutaneous air pouch (SAP), with measurement of cytokines. The results showed antinociceptive effect for ZPEO for the first phase of the formalin-induced licking, glutamate, and hot plate tests. However, ZPEO had no effect on reducing paw licking induced by capsaicin. Finally, ZPEO had no effect against inflammation induced by carrageenan.

## 1. Introduction

Essential oils are naturally occurring complex molecules composed mainly of monoterpenes. They have been used in several industries around the world, especially for cosmetics including beauty creams and perfumes due to their pleasant scents. Essential oils have also been used to treat several diseases and some of them have been tested for medicinal purposes, such as treating pain and inflammation [1].

Known in Japan as Asakura sansho,* Zanthoxylum piperitum* (ZP) first attracted the attention of researchers due to its aroma [2, 3]. Later, the research became more focused on its antioxidative effect resulting in very positive outcomes contributing to the cosmetic industry. In 2001, Hashimoto et al. [4] reported the ability of an aliphatic acid from ZP in inducing relaxation in the circular muscle of the gastric body.

Perhaps due to its promising results as an antioxidant, ZP was also tested for anti-inflammatory activities targeting nitric oxide and cytokines production. This treatment was made from fresh and dried fruits of ZP and showed an inhibitory effect on cytokines (TNF-*α* and IL-1*β*) production from mouse macrophage cells [5]. The anti-inflammatory effect correlated with the production of nitric oxide was also described after testing ZPEO. The essential oil also had an effect on reduction of cyclooxygenase-2 expression and activity. Later, a glycoprotein (24 kDa) was isolated from ZP fruits to investigate its anti-inflammatory potential. It was shown to suppress cytokines IL-1*β*, IL-6, and TNF-*α* production and expression of inducible nitric oxide synthase (iNOS), COX-2, and myeloperoxidase 9 (MMP-9) [6]. This glycoprotein was reported to prevent inflammatory gastrointestinal diseases [7], and a larger glycoprotein (115 kDa) was also effective in blocking proinflammatory signals [8].

The strong correlation between antioxidant compounds also having anti-inflammatory activities was later reported by Diaz et al. [9]. Furthermore, substances that are antioxidant and anti-inflammatory have been reported to be likely to have an anticancer effect [10]. This anticancer effect was first described in relation to ZP by the Japanese group of Hirokawa et al. [11], suggesting that ZP extract could potentially be useful against breast cancer.

ZP was also tested as part of a herbal formulation for periodontitis showing substantial improvement especially in recovery of collagen gingival tissue [12]. In Korea ZP is used in traditional medicine as a diuretic and to treat digestive disorders. It is also used to help the cardiovascular system [12]. Some ZP compounds have been described to inhibit cholesterol acetyltransferase, thus contributing to helping the cardiovascular system, which validates the traditional use since cholesterol ester plays an important role in cardiovascular diseases [13]. The fact that ZP is used for digestive disorders could indicate an effect on stomach pain that might suggest pain relief properties for this species.

As part of our continuous interest in search for pharmacological effect of natural products and because ZP is widely used to treat several disorders, in this work we focused our efforts on the evaluation of the possible antinociceptive effect of essential oil obtained from* Zanthoxylum piperitum*.

## 2. Material and Methods

### 2.1. Plant Material

Plant material (aerial parts) of* Z. piperitum* was collected from the Glasnevin Botanic Gardens, Dublin, and dried at room temperature for two weeks. Dr. Colin Kelleher from the Glasnevin Botanic Gardens identified the species and a herbarium sample is kept in the Botanic Garden under the collection number 1984.1920.

### 2.2. Isolation of the Essential Oil

Air-dried, to constant weight, plant material (3 batches of 250 g of aerial parts) was subjected to hydrodistillation with circa one liter of distilled H_2_O for 2.5 h using the original Clevenger-type apparatus. The yield was 0.015% (w/w, dried weight basis) for the oil isolated from* Z. piperitum* (ZPEO). The obtained oil was separated by extraction with Et_2_O (Merck, Germany), dried over anhydrous Na_2_SO_4_ (Aldrich, USA), and immediately analysed.

### 2.3. Chemical Analysis of ZPEO

Qualitative analyses were carried out on a GC-QP2010 PLUS Shimadzu with a ZB-5MS fused silica capillary column (30 m × 0.25 mm × 0.25 *μ*m film thickness) under the experimental conditions reported for GC-FID analysis. The essential oil components were identified by comparing their retention indices and mass spectra to published data and computer matching with WILEY 275 and the National Institute of Standards and Technology (NIST 3.0) libraries provided by a computer-controlled GC-MS system. The results were also confirmed by comparing the compounds' elution order with their relative retention indices reported in the literature [14]. The retention indices were calculated for all the volatile constituents using the retention data of linear* n*-alkanes C8–C24.

### 2.4. Animals

Swiss Webster mice (20–25 g, two months old), donated by Instituto Vital Brazil (Niterói, Rio de Janeiro, Brazil), were used in this study. The animals were maintained in standard conditions (room with light-dark cycle of 12 h, 22 ± 2°C to 70% to 80% humidity, and with food and water* ad libitum*). Twelve hours before assays the animals were maintained in fasting in order to avoid food interference with the absorption of the tested substances. Animals were acclimatized to the laboratory conditions for at least 1 h before each test on set and were used only once throughout the experiments. All protocols were conducted in accordance with the Guidelines on Ethical Standards for Investigation of Experimental Pain in Animals and followed the principles and guidelines adopted by the National Council for the Control of Animal Experimentation (CONCEA), approved by the Biomedical Science Institute/UFRJ, Ethical Committee for Animal Research, and received the number DFBCICB015-04/16. All experimental protocols were performed during the light phase. Animal numbers per group were kept at a minimum and according to rules from CONCEA. At the end of each experiment mice were killed by ketamine/xylazine overdose.

### 2.5. Formalin-Induced Acute Pain

Twenty microliters of 2.5% formalin (37% formaldehyde) was injected in the plantar region of the right hind paw of mice 30 min after oral treatment with ZPEO (10, 30  or 100 *μ*L/kg) or vehicle (oil) or 1 hour after oral treatment with morphine (2.5 mg/kg) or acetylsalicylic acid (200 mg/kg). The animals were placed individually in a transparent glass chamber and the duration of time (in seconds) that they spent licking their paw after injection of formalin was recorded and analysed over two separate periods: 0–5 minutes after injection (named early phase or neurogenic pain) and 15–30 minutes after injection (named late phase or inflammatory pain).

### 2.6. Capsaicin-Induced Nociception

This test was based on the method described by Sakurada et al. [15] with some modifications. Capsaicin (1.6 *μ*g/paw) was injected into the plantar region of the right hind paw of the mice one hour after treatment. The animals were placed individually in a transparent glass chamber and paw licking duration (seconds) was recorded (0–5 minutes after capsaicin injection) and analysed.

### 2.7. Glutamate-Induced Nociception

This method was first described by Beirith et al. [16]. One hour after oral treatment of ZPEO, the plantar region of the right hind paw of the mice was injected with 20 *μ*L of glutamate solution in PBS (3.7 ng/paw). The animals were placed individually in a transparent glass chamber and paw licking duration (seconds) was recorded (0–15 minutes after glutamate injection) and analysed.

### 2.8. Central Nociception: Hot Plate Test

This test was based on the method described by Ohlsson [17]. Mice were treated with ZPEO (10, 30, or 100 *μ*L/kg, p.o.), vehicle (oil), or morphine (2.5 mg/kg, p.o.). They were placed on the hot plate apparatus (Insight, Brazil), kept at a constant temperature of 55 ± 0.5°C. The latency time until the animal began jumping, licking, or shaking the paw was recorded. The measurements occurred before treatment (baseline, mean of 60 and 30 minutes before treatment) and 30, 60, 90, 120, and 180 minutes after treatment. In order to prevent tissue damage to paw, a maximum exposure time (cut-off) of the animal's paws to the heated plate was established.

Aiming to investigate the antinociceptive mechanism involved, the animals were treated intraperitoneally 15 minutes before the oral treatment with ZPEO, with naloxone (a nonselective antagonist of the opioid receptor, 1 mg/kg) or atropine (a nonselective antagonist of the muscarinic receptor, 1 mg/kg).

The results for the hot plate test were expressed as a percentage increase compared to baseline (% ICB), calculated by the formula latency × 100/baseline − 100 and area under the curve.

### 2.9. Acute Toxicity

To exclude a possible toxicity in bone marrow and circulating leukocytes 24 hours after treatment with 100 *μ*L/kg of ZPEO mice were anesthetized with ketamine/xylazine, blood was collected by orbital plexus into a heparinized tube, and after that mice were euthanized. The bone marrow was collected from the animal's femur by washing with 1 mL of PBS into the cavity. Haemogram analysis was performed in a CellPocH-100iV Diff (Sysmex) hematology analyser.

### 2.10. Inflammation Model: Subcutaneous Air Pouch (SAP) Model

This model was described by Sedgwick et al. [18] with modifications done by Raymundo and colleagues [19]. The animals received a dorsal subcutaneous injection of sterile air (10 mL) and an addition of 7 mL of air on the third day. On the sixth day, animals received a subcutaneous injection of sterile carrageenan solution (1%; 1 mL). Mice were pretreated with vehicle or the ZPEO (10, 30, or 100 *μ*L/kg) 1 h before carrageenan injection into the SAP. The control group received an injection of sterile PBS (1 mL) into the SAP. Animals were sacrificed 24 h after carrageenan injection. The cavity was washed with 1 mL of PBS and the exudates were collected. The total cell counts were carried out from the exudates using a CellPocH-100iV Diff (Sysmex) hematology analyser. The exudates were centrifuged at 12,000 rpm for 8 min at 4°C and aliquots of the supernatants were stored at −20°C for dosages of tumour necrosis factor-*α* (TNF-*α*) and extravasated protein. TNF-*α* dosage was carried out by enzyme-linked immunosorbent assay (ELISA) according to the manufacturer's instructions (B&D, USA). Extravasated protein was determined using the BCA method (BCA*™* Protein Assay Kit, Pierce). The results are expressed as pg/mL of TNF-*α* or mg/mL of protein.

### 2.11. Statistical Analysis

All experimental groups consisted of 6–10 mice. The results are presented as the mean ± SD. Statistical significance between groups was performed by applying analysis of one-way variance (ANOVA) followed by Dunnett's and Bonferroni's test using* GraphPad Prism 5.0 *software. *p* values less than 0.05 (*p* < 0.05) were considered significant.

## 3. Results

**Figure 1:**
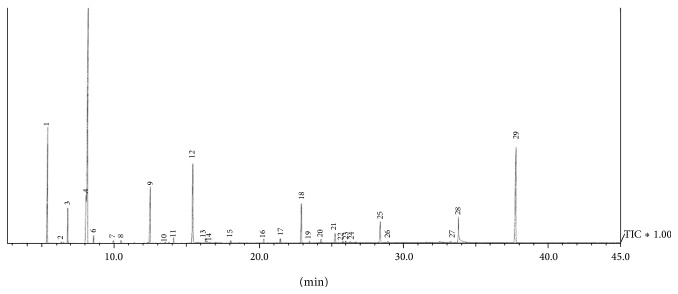
Gas chromatogram of* Z. piperitum* essential oil.

*GC-FID and GC/MS Analyses.* Analysis of the essential oil was carried out by GC and GC/MS. The GC/MS analyses (three repetitions) were performed on a GC-QP2010 PLUS Shimadzu with a ZB-5MS fused silica capillary column (30 m × 0.25 mm × 0.25 *μ*m film thickness) and coupled with a 5975B mass-selective detector from the same company. The injector and interface were operated at 260° and 200°, respectively. The oven temperature was raised from 60 to 240° at a heating rate of 3° min^−1^ and then isothermally held for 10 min. As a carrier gas, He at 1.0 mL min^−1^ was used. The sample, 1 mL of the solutions, in Et_2_O (10 mg in 1 mL of Et_2_O), was injected in a pulsed split mode (the flow was 1.5 mL/min for the first 0.5 min and was then set to 1.0 mL/min throughout the remainder of the analysis; split ratio 40 : 1). The mass-selective detector was operated at the ionization energy of 70 eV, in the 35–650 amu range and a scanning speed of 3 scans/sec. GC (FID) analysis was carried out under the same experimental conditions using the same column and the same gas chromatograph type as described for the GC/MS. The percentage composition was computed from the total ion chromatogram peak areas without the use of correction factors. Qualitative analysis was based on the comparison of their linear retention indices relative to retention times of C8–C24* n*-alkanes on the DB-5MS column with those reported in the literature [14] and by comparison of their mass spectra with those from Wiley 6, NIST07, MassFinder 2.3 (Figure 1 and Table 1).

### 3.1. Effect of* Z. piperitum* on Formalin-Induced Acute Pain

**Figure 2:**
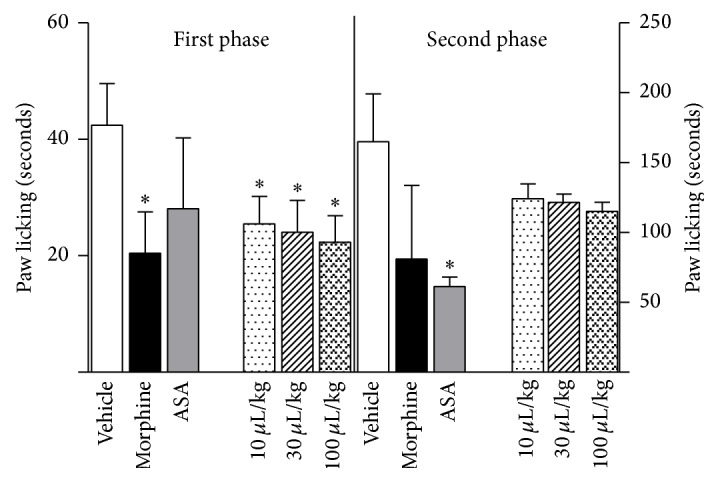
Effect of essential oil from* Zanthoxylum piperitum* DC. on formalin-induced licking in mice (first and second phases). Animals were pretreated with ZPEO (10, 30, or 100 *μ*L/kg, p.o.), acetylsalicylic acid (ASA, 200 mg/kg, p.o.), morphine (2.5 mg/kg, p.o.), or vehicle (oil). Results are presented as mean ± SD (*n* = 6–10). Statistical analyses were performed using GraphPad Prism version 5.1 software, by one-way ANOVA with Dunnett's posttest with multiple comparisons against vehicle-treated group (^*∗*^ *p* < 0.05).

In the formalin-induced acute pain test, all doses (10, 30, and 100 *μ*L/kg) were able to decrease paw licking in the first phase of the test. The doses reduce by 28%, 34%, and 43.3% the licking time, respectively. However, ZPEO did not decrease licking in the second phase of the response to formalin injection (Figure 2).

### 3.2. Effect of* Z. piperitum* on Glutamate-Induced Nociception

**Figure 3:**
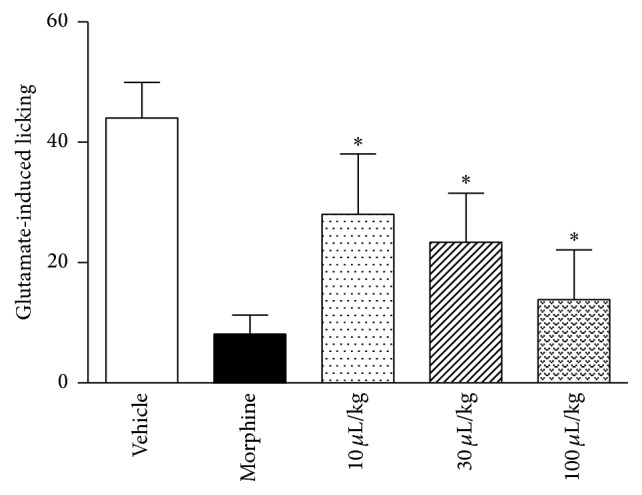
Effect of essential oil from* Zanthoxylum piperitum* DC. on glutamate-induced licking in mice. Animals were pretreated with ZPEO (10, 30, or 100 *μ*L/kg, p.o.), morphine (2.5 mg/kg, p.o.), or vehicle (oil). Results are presented as mean ± SD (*n* = 6–10). Statistical analyses were performed using GraphPad Prism version 5.1 software, by one-way ANOVA with Dunnett's posttest with multiple comparisons against vehicle-treated group (^*∗*^ *p* < 0.05).

ZPEO decreased paw licking induced by glutamate at a dose of 10, 30, and 100 *μ*L/kg (25%, 39%, and 64%, resp.). The standard drug morphine presented a 79% reduction of the glutamate paw licking (Figure 3).

### 3.3. Effect of* Z. piperitum* on Capsaicin-Induced Nociception

**Figure 4:**
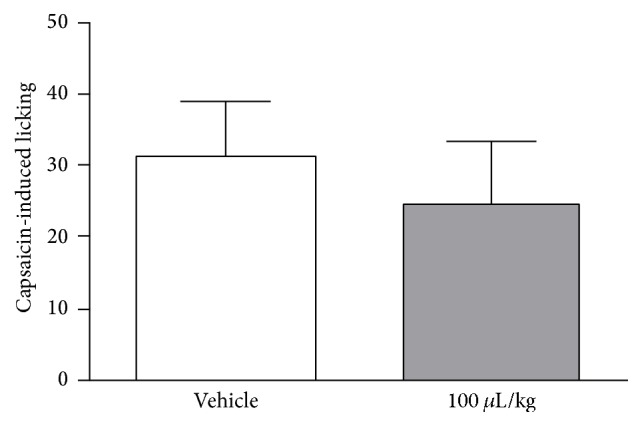
Effect of essential oil from* Zanthoxylum piperitum* DC. on capsaicin-induced licking in mice. Animals were pretreated with ZPEO (100 *μ*L/kg, p.o.) or vehicle (oil). Results are presented as mean ± SD (*n* = 6–10). Statistical analyses were performed using GraphPad Prism version 5.1 software, by one-way ANOVA with Dunnett's posttest with multiple comparisons against vehicle-treated group.

In order to verify if ZPEO would interfere with TRPV1 receptors, it was tested in a model of pain induced by capsaicin. The result of this evaluation showed that oral administration of ZPEO (100 *μ*L/kg) was unable to decrease licking induced by capsaicin (Figure 4).

### 3.4. Effect of* Z. piperitum* on Thermal Nociception

**Figure 5:**
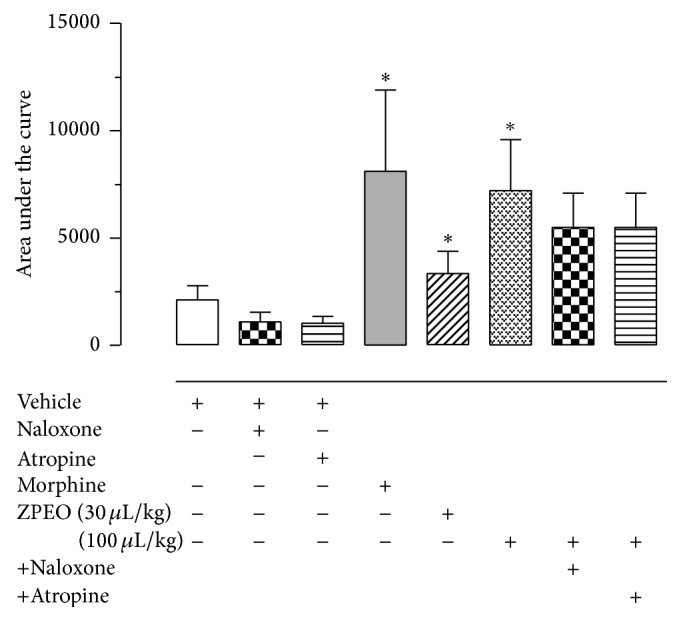
Effect of essential oil from* Zanthoxylum piperitum* DC. on thermal nociception (hot plate test). Animals were orally pretreated with ZPEO (30 or 100 *μ*L/kg), morphine (2.5 mg/kg), or vehicle (oil). Results are presented as mean ± SD (*n* = 6–10) of area under the curve. Statistical analyses were performed using GraphPad Prism version 5.1 software, by one-way ANOVA with Dunnett's posttest with multiple comparisons against vehicle-treated group (^*∗*^ *p* < 0.05).

Through the hot plate test, which measures central antinociception, ZPEO was only able to increase animal paw withdrawal threshold when treated with a higher dose of 100 *μ*L/kg. The lower dose of 30 *μ*L/kg did not show activities. The treatment of the animals with either naloxone, a nonselective antagonist of the opioid receptor, or atropine, a nonselective antagonist of the muscarinic receptor, did not have any effect on that antinociception (Figure 5).

### 3.5. Effect of* Z. piperitum* on Leukocytes Migration into Subcutaneous Air Pouch (SAP)

**Figure 6:**
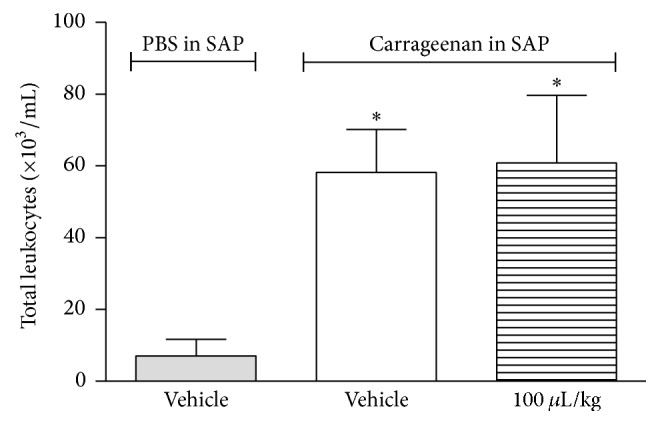
Effect of ZPEO on leukocytes migration through SAP model. Animals were pretreated with ZP (100 *μ*L/kg, p.o.) 1 h prior to carrageenan (1%) injection into the SAP. The group vehicle received either carrageenan (1%) or PBS injected into the SAP. Results are presented as mean ± SD (*n* = 6–10) of total leukocytes (×10^3^/mL). Statistical analyses were performed using GraphPad Prism version 5.1 software, by one-way ANOVA with Dunnett's posttest with multiple comparisons against vehicle-treated group. ^*∗*^ *p* < 0.05 when comparing to vehicle-treated group.

**Figure 7:**
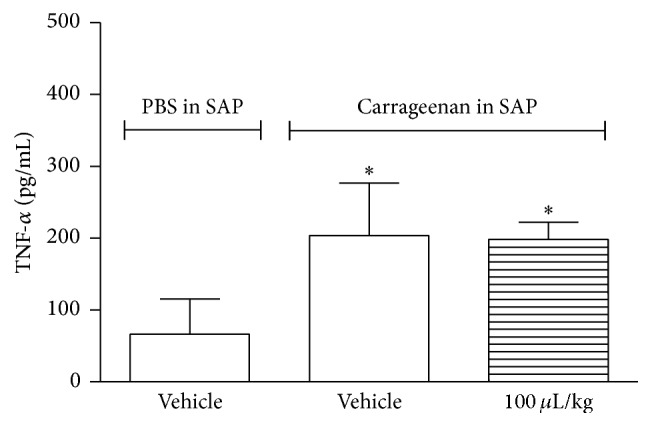
Effect of ZPEO on TNF-*α* production in the subcutaneous air pouch (SAP) model. Animals were pretreated with ZPEO (100 *μ*L/kg, p.o.) 1 h prior to carrageenan (1%) injection into the SAP. The group vehicle received either carrageenan (1%) or PBS injected into the SAP. Results are presented as mean ± SD (*n* = 6–10) of TNF-*α* (pg/mL). Statistical analyses were performed using GraphPad Prism version 5.1 software, by one-way ANOVA with Dunnett's posttest with multiple comparisons against vehicle-treated group. ^*∗*^ *p* < 0.05 when comparing to vehicle-treated group.

**Figure 8:**
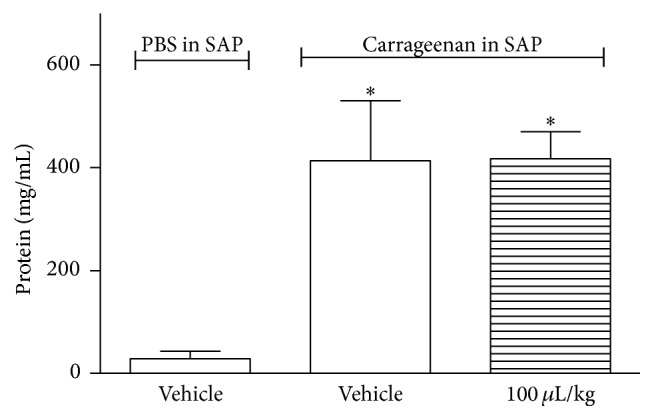
Effect of ZPEO on protein extravasation in the subcutaneous air pouch (SAP) model. Animals were orally pretreated with ZPEO (100 *μ*L/kg) 1 h prior to carrageenan (1%) injection into the SAP. The group vehicle received either carrageenan (1%) or PBS injected into the SAP. Results are presented as mean ± SD (*n* = 6–10) of protein (mg/mL). Statistical analyses were performed using GraphPad Prism version 5.1 software, by one-way ANOVA with Dunnett's posttest with multiple comparisons against vehicle-treated group. ^*∗*^ *p* < 0.05 when comparing to vehicle-treated group.

ZPEO did not reduce the number of leukocytes that migrate to the SAP after carrageenan injection (Figure 6) nor did it have an effect on TNF-*α* production by leukocytes and on extravasation of protein (Figures 7 and 8).

### 3.6. Acute Toxicity

Twenty-four hours after oral administration of ZPEO (100 *μ*L/kg) aliquots of blood and femoral fluxes were obtained and leukocyte counts were performed. The results indicated that the essential oil did not affect leukocyte number either in bone marrow or in blood (data not shown).

## 4. Discussion

The formalin test is believed to be a test that closely simulates clinical pain because it generates injured tissue. The test is known to induce phases and predominately involves C fibres. The early phase of this test has participation of substance P and bradykinin while the later phase also known as inflammatory pain phase has involvement of histamine, serotonin, prostaglandin, and again bradykinin [20]. In this study, ZPEO was able to decrease paw licking in the first phase but not in the second phase. The fact that bradykinin appears in both phases could indicate that the effect observed is not through bradykinin interference.

More recent publications indicate the participation of glutamate in both phases of the formalin test and a special participation of adenosine A_2_ NMDA glutamate receptor binding in the first phase of the formalin test [21]. Interestingly, in the present research, ZPEO was effective in reducing nociception in the glutamate-induced licking test even in the lower dose of 10 *μ*L/kg. This result suggests that the ZPEO could act on glutamatergic pathways and could involve the same NMDA receptor. An antagonist of this receptor could be used in future studies to check this. On the other hand, only the higher dose 100 *μ*L/kg had an effect in the hot plate test, which measures central nociception activity and which mechanism involves mainly A*δ* fibres but also has involvement of glutamate receptors. Drugs such as morphine and oxotremorine are, respectively, opioid and muscarinic receptor agonists, and they also have an antinociceptive effect in the hot plate test [22]. The use of the opioid and cholinergic antagonists did not decrease the antinociception observed for ZPEO. Therefore, this suggests that the effect of ZPEO observed in the hot plate test is mediated through neither the opioid nor the muscarinic receptors. For this reason, it is very likely that the effect of ZPEO observed in both models is due to the ability of ZPEO compound(s) to inhibit the excitatory transmission induced by glutamate. This effect is also observed using morphine and as shown in the results this drug has an effect in the hot plate test and a potent effect in the first phase of the formalin- and glutamate-induced licking tests. Nevertheless, the fact that ZPEO antinociception is not mediated through the opioid receptor makes it an ideal candidate as a potential analgesic, since medicines that have an effect through this mechanism have been shown to be more likely to cause side effects such as tolerance, hyperalgesia, and drug dependence [23].

ZPEO did not reduce capsaicin-induced licking. This result indicates that the compounds in ZPEO are not having an effect on TRPV1 receptors, although this receptor is also playing an important role in the hot plate models and its connection with glutamatergic pathway [24].

In this present study ZPEO had no effect on inflammation inducing mediators such as TNF-*α*. The anti-inflammatory effect of ZPEO has been reported by several research groups. Reference [25] showed ZPEO effect on cyclooxygenase, an enzyme involved in prostaglandin production and which is an inflammatory mediator. Regarding mediators, Yang et al. [26] reported ZP effect on TNF-*α* using ZP compounds isolated from pericarps; these compounds were ZP amides (A, B, D, and F), bungeanumamide A, tumuramide C, hypericin, sesamin, and quercitrin. Also, the ability of suppressing TNF-*α*, interleukin- (IL-) 1*β*, and interleukin-6 was reported earlier by Kim et al. [27]. The major compounds described in that research were octanoic acid (13.4%),* n*-heptanol (9.8%), and 1-octanol (8.1%). None of these compounds were present in the ZPEO; perhaps this could explain the inability of ZPEO as an anti-inflammatory agent.

The toxicity of ZP has been looked into by other researchers and it was found that it is phytotoxic due to the presence of the compound eucarvone [28]. The cell death occurred due to overproduction of reactive oxygen species (ROS). Eucarvone was not present in ZPEO analysed in the present study. On the other hand, Lee et al. [6] reported the antioxidant and protective effect against hepatotoxicity for glycoproteins from ZP. This protective effect is obtained by inducing apoptosis and enhancing the activity of natural killer cells [29]. Therefore, the importance of chemical identities when testing ZP essential oils is emphasised.

## 5. Conclusions

The essential oil of* Z. piperitum* has a significant antinociceptive activity. This effect is not mediated through opioid or muscarinic receptors. However, it seems to have interference on the glutamatergic pathway. These findings suggest that* Z. piperitum* essential oil containing the chemical profile presented in this study has potential as an analgesic medicine and the fact that it is not mediated through the opioid mechanism suggests that it is less likely to cause serious side effects.

## Figures and Tables

**Table 1:** Chemical constituents identified in ZPEO. See Figure 1.

ZPEO	RT	%	RI Lit	RI
(1) Alpha-pinene	5.33	9.75%	933	930
(2) Sabinene	6.23	0.12%	976	971
(3) Beta-myrcene	6.75	3.34%	991	987
(4) Limonene	8.02	5.48%	1031	1028
(5) *β*-Phellandrene	8.08	29.39%	1031	1030
(6) *cis*-*β*-ocimene	8.54	0.79%	1040	1044
(7) Terpinolene	9.91	0.30%	1088	1084
(8) *β*-Linalool	10.43	0.33%	1097	1099
(9) *β*-Citronellal	12.43	6.83%	1153	1151
(10) *β*-Terpineol	13.52	0.14%	1159	1179
(11) *α*-Terpineol	14.10	0.72%	1189	1194
(12) *β*-Citronellol	15.39	10.32%	1228	1226
(13) *cis*-Geraniol	16.31	0.64%	1252	1249
(14) Piperitone	16.41	0.24%	1255	1251
(15) 2-Decanone	18.04	0.33%	1292	1291
(16) Citronellyl acetate	20.32	0.57%	1354	1348
(17) Neryl acetate	21.45	0.57%	1365	1377
(18) Caryophyllene	22.89	5.56%	1419	1413
(19) *α*-Bergamotene	23.49	0.22%	1436	1429
(20) *α*-Humulene	24.27	0.51%	1455	1449
(21) Germacrene D	25.25	1.38%	1480	1475
(24) E,E-*α*-farnesene	26.29	0.16%	1508	1502
(25) *trans*-Nerolidol	28.38	2.95%	1563	1520
(28) (E,E)-Farnesol	33.83	3.98%	1722	1711
(29) (E,E)-Farnesyl acetate	37.78	14.55%	1843	1830

Total % of components identified: 99.17%.
